# Nerve growth factor for Bell’s palsy: A meta-analysis

**DOI:** 10.3892/etm.2014.2100

**Published:** 2014-12-03

**Authors:** YIPENG SU, XIAOMENG DONG, JUAN LIU, YAOZHI HU, JINBO CHEN

**Affiliations:** 1 Binzhou Medical University, Yantai, Shandong 264003, P.R. China; 2 Department of Neurology, The Affiliated Hospital of Binzhou Medical University, Binzhou, Shandong 256603, P.R. China; 3 Library of Binzhou Medical University, Binzhou, Shandong 256603, P.R. China

**Keywords:** nerve growth factor, Bell’s palsy, meta-analysis

## Abstract

A meta-analysis was performed to evaluate the efficacy and safety of nerve growth factor (NGF) in the treatment of Bell’s palsy. PubMed, the Cochrane Central Register of Controlled Trials, Embase and a number of Chinese databases, including the China National Knowledge Infrastructure, China Biology Medicine disc, VIP Database for Chinese Technical Periodicals and Wan Fang Data, were used to collect randomised controlled trials (RCTs) of NGF for Bell’s palsy. The span of the search covered data from the date of database establishment until December 2013. The included trials were screened comprehensively and rigorously. The efficacies of NGF were pooled via meta-analysis performed using Review Manager 5.2 software. Odds ratios (ORs) and 95% confidence intervals (CIs) were calculated using the fixed-effects model. The meta-analysis of eight RCTs showed favorable effects of NGF on the disease response rate (n=642; OR, 3.87; 95% CI, 2.13–7.03; P<0.01; I^2^=0%). However, evidence supporting the effectiveness of NGF for the treatment of Bell’s palsy is limited. The number and quality of trials are too low to form solid conclusions. Further meticulous RCTs are required to overcome the limitations identified in the present study.

## Introduction

Bell’s palsy, also known as idiopathic facial nerve paralysis (IFNP), is an acute, idiopathic and unilateral paralysis of the face. This common condition affects 20–45 cases per 100,000 people (1). At present, although the aetiology remains unknown, autoimmune disorders and inflammations due to viral infections are considered to play two key roles in the development of the disease (2–4). The prognosis of the disease is favourable, and 70–80% of all patients with IFNP are likely to have complete or near complete recovery, regardless of whether treatment is given (5). However, poor recovery of facial muscle control, facial disfigurement, psychological trauma and facial pain are likely to affect <30% of patients.

Improving the recovery of facial functions and preventing associated complications are the priorities in treatment. During the acute stage of the disease, corticosteroid and antiviral medications have been proved to be effective treatments (6,7). During the sequelae stage, botulinum toxin A and surgical reconstruction are the treatment options for facial spasms and synkinesis. Physical therapy and acupuncture are also used to treat Bell’s palsy (8).

Nerve growth factor (NGF) was the first member of the neurotrophin family to be identified (9). Neurotrophins are critical to the development and maintenance of the central and peripheral nervous systems. They prevent or reverse neuronal degeneration, promote neurite regeneration and enhance synaptic plasticity (10,11). Given its potential roles, NGF has been widely used in neurological disorders, particularly in China. Three brands of NGF drug are available in China. A number of doctors use the drug as an adjuvant treatment for peripheral neuropathy, including Bell’s palsy (12). To date, however, no systematic review has provided evidence on the efficacy of NGF for Bell’s palsy. Clearly, the efficacy and safety of NGF should be strictly assessed prior to being recommended for routine use in patients with Bell’s palsy. Therefore, we conducted a systematic review to critically evaluate the effect of NGF treatment on Bell’s palsy.

## Materials and methods

### Search sources and strategy

The following electronic databases were searched, starting from their inception to December 2013: PubMed, the Cochrane Central Register of Controlled Trials, Embase, the China National Knowledge Infrastructure, China Biology Medicine disc, VIP Database for Chinese Technical Periodicals and Wan Fang Data. The search strategy combined the following specific Medical Subject Heading and free-text words using the following terms: Bell’s palsy, facial paralysis, idiopathic facial palsy, herpetic facial paralysis and nerve growth factor or NGF. In addition, manual searches were performed in specialised journals. No restriction was applied on publication date or language.

### Study selection and inclusion/exclusion criteria

Identified trials were evaluated independently by two of the co-authors of the present study, Dr Yipeng Su (SYM) and Dr Xiaomeng Dong (DXM), following a defined protocol. The inclusion criteria were as follows: i) The study design had to be a randomised controlled trial (RCT); ii) the study had to include patients with unilateral facial nerve weakness with no identifiable cause observed within seven days of the onset of weakness; iii) treatment had to start within seven days of paralysis onset; and iv) the follow-up had to last at least four weeks. By contrast, studies that satisfied the following criteria were excluded: i) Any study that included patients with suppurative otitis media, multiple sclerosis, traumatic facial paralysis, encephalitis and Lyme disease; ii) any study that included children or pregnant or breastfeeding females; iii) any study where the study design involved animals, case reports, case series, retrospective studies or cohort studies; and iv) redundant studies or duplicate publications. If a study did not provide the necessary information to assess potential eligibility, then the authors were contacted and asked to provide the missing data.

### Data extraction and quality assessment

The following variables were extracted from all studies: General characteristics (type of study, year of publication, number of patients included and their baseline characteristics); procedural data (type of randomisation, inclusion criteria, NGF dose, follow-up protocol and number of patients who dropped out of the study) and outcome data (definition for facial recovery and facial muscle recovery outcome, and adverse effects of each therapy strategy). Any disagreement was resolved by discussion.

Two independent investigators (SYP and DXM) evaluated study quality. The modified Jadad score (13) was used to assess study quality. This score considers randomisation technique, allocation concealment, blinding, intention to treat and patient attrition. Studies with a score of 4–7 were considered to be of high quality, whereas those with a score of 0–3 were considered of poor quality. Any disagreement was resolved by discussion.

### Primary outcome

The primary outcome of the meta-analysis was the proportion of patients who attended a follow-up visit at least four weeks after treatment initiation, with at least partial facial muscle recovery. Complete recovery was defined as a score of ≤2 on the House-Brackmann Facial Recovery (H-B) scale (14–16). Partial recovery was defined as a score of 3–6 according to H-B scale. The secondary outcome was the range of recovery of the compound muscle action potential (CMAP) of the facial nerve measured by electroneurography (ENoG).

### Statistical analysis

To summarise the effects of NGF on disease response rate, the risk estimates [odds ratio (OR) and mean difference (MD)] and 95% confidence interval (CI) from each study were calculated using the Cochrane Collaboration’s Review Manager 5.2 software (The Nordic Cochrane Centre, Copenhagen, Denmark). The heterogeneity of ORs was assessed using Cochrane’s Q test and I^2^. If heterogeneity was present, the ORs were pooled using the random-effects model (the DerSimonian and Laird method). Otherwise, the fixed-effects model (the Mantel-Haenszel method) was used. The statistical significance of the pooled ORs and MDs was analysed using the Z test. A funnel plot was used to detect publication bias. For studies with insufficient information, the authors of the present study contacted the primary authors of the studies undergoing analysis to acquire and verify data whenever possible.

## Results

### Study description and quality

**Figure 1:**
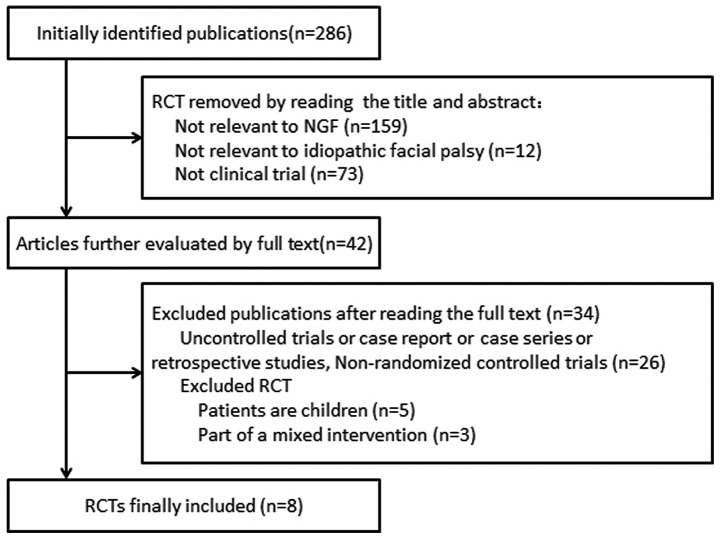
Flow chart of the trial selection process. RCT, randomised controlled trial; NGF, nerve growth factor.

The literature search from the various databases identified 286 relevant studies. From this pool of relevant studies, eight (17–24) met our inclusion criteria (Fig. 1). All trials originated in China. The characteristics of the eight studies are summarised in Table I. All the participants in these studies suffered from Bell’s palsy. The total number of participants was 642 (NGF group, n=317 and Control group, n=325), all of whom were adults, excluding pregnant and breastfeeding females. All trials employed a two-arm parallel group design.

The specific drug and dosage used for all trials are provided in Table I. All trials employed the recovery of facial nerve motor function as the main outcome measure based on the H-B scale. In two studies, ENoG was used to evaluate the effect of NGF on facial nerve recovery; the CMAP of the facial nerve was also measured. Four studies (17–19,24) reported adverse events during the trial. However, adverse events such as gastrointestinal disorders were likely caused by corticosteroids rather than NGF.

The majority of the RCTs included in this review had a high risk of bias. Only two RCTs employed an appropriate sequence-generation method (21,22). None of the studies reported allocation concealment or blinding. Table I provides the results of the assessment conducted on the quality of the included trials.

### Meta-analysis

**Figure 2:**
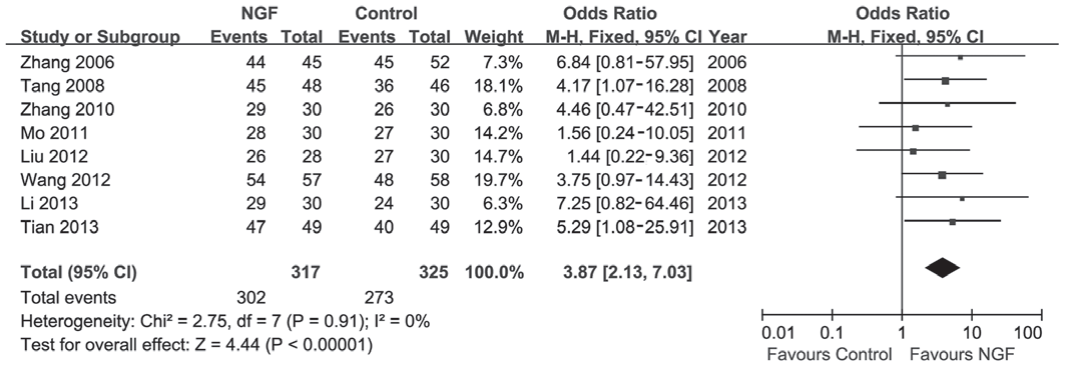
Forest plot of NGF for Bell’s palsy on response rate by the House-Brackmann Facial Recovery scale. NGF, nerve growth factor; M-H, Mantel-Haenszel method; CI, confidence interval.

All the RCTs indicated the number of participants who responded to the therapy. As shown in Fig. 2, moderate heterogeneity was observed among trials (P=0.91; I^2^=0%). A meta-analysis was conducted using the fixed-effect model. The result of the meta-analysis showed favourable effects of NGF on response rate (n=642; OR, 3.87; 95% CI, 2.13–7.03; P<0.01; Fig. 2).

**Figure 3:**
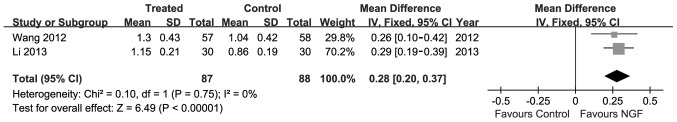
Forest plot of NGF for Bell’s palsy on response rate by electroneurography. SD, standard deviation; NGF, nerve growth factor; CI, confidence interval; IV, inverse variance method.

For the secondary outcome, i.e., facial nerve CMAP, the meta-analysis also showed the favourable effects of NGF on response rate. Heterogeneity was also observed between two trials (P=0.75; I^2^=0%), and the fixed-effect model was used to perform the meta-analysis (n=175; MD, 0.28; 95% CI, 0.2–0.37; P<0.01; Fig. 3).

**Figure 4:**
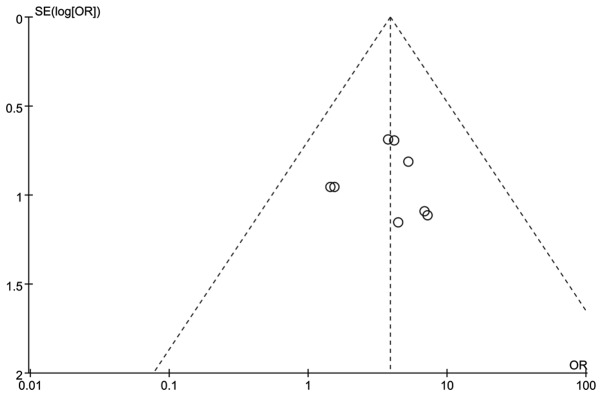
Funnel plot for the eight studies concerning nerve growth factor for Bell’s palsy. SE, standard error of the mean; OR, odds ratio.

A funnel plot was constructed based on the comparison of the total efficacy rate of two groups (NGF versus Control), and the presence of publication bias was visually assessed. The resultant funnel shape was almost symmetrical (Fig. 4), thus indicating that no potential publication bias occurred.

**Figure 5:**
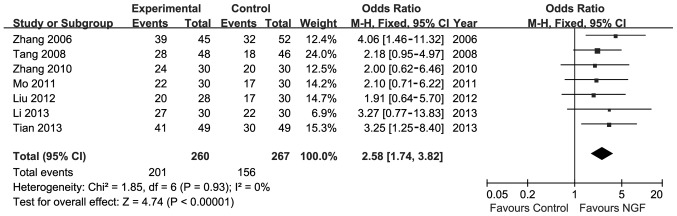
Sensitivity analysis of NGF for Bell’s palsy on response rate. NGF, nerve growth factor; M-H, Mantel-Haenszel method; CI, confidence interval.

Sensitivity analysis was conducted by substituting complete recovery for partial recovery as the primary outcome. The meta-analysis showed significant improvements in the NGF group (n=527; OR, 2.58; 95% CI, 1.74–3.82; P<0.01; I^2^=0%; Fig. 5). Risk ratio was also used instead of OR, and the data did not change relative to the outcomes.

## Discussion

Bell’s palsy is an idiopathic peripheral nerve palsy that involves the facial nerve and is a common peripheral neuropathy worldwide. Although prognosis is favorable, <30% of patients are likely to experience sequelae and the recovery period can last for more than two months. At present, corticosteroid and anti-viral therapy are the most effective treatments (6,7). Reports on the use of NGF to treat Bell’s palsy are lacking, and few trials have examined the effects of NGF treatment on Bell’s palsy. The results from the limited number of studies showed a more favourable effect when NGF was used as an adjunct to conventional drug treatment compared with conventional drug treatment alone (17–24). However, the number of trials is small and the risk of bias is too high to draw any solid conclusions. The findings of the present study provide limited evidence that NGF is beneficial to the symptomatic treatment of Bell’s palsy.

A strong attempt was made in the present study to search the available literature comprehensively. However, there cannot be absolute certainty that all the relevant articles were identified. Furthermore, selective publishing is another major cause of bias. NGF drug has not been used in the USA or Europe; thus, the majority of the clinical studies on NGF were conducted in China. As such, existing potential regional bias is an issue. All the available trails exhibited the positive effects of NGF in treating Bell’s palsy. Considering that no other systematic study of NGF as a facial palsy treatment is available, it was not possible to compare the results of the present study with other meta-analyses. The paucity and sub-optimal methodological quality of the primary data create further shortcomings in the available evidence, thereby markedly limiting the conclusiveness of this systematic review (25).

Of the eight RCTs included in the present meta-analysis, all the trials compared NGF with conventional drug therapy versus conventional drugs only. This type of pragmatic design may generate favourable effects on at least one outcome measure. Considering this design, the aforementioned trials are unable to demonstrate specific therapeutic effects (26).

Since 1986, when Levi-Montalcini and Cohen won the Nobel Prize in Physiology or Medicine for their work on NGF (27,28), numerous pharmaceutical companies and research institutions have begun to identify the mechanisms of action and protective effects of NGF. Assuming that NGF is beneficial in the treatment of Bell’s palsy, possible mechanisms of action are of interest. NGF has an important role in the neurorepair process following the occurrence of damage (29). One previous study (30) arrived at the same conclusion subsequent to using NGF to treat traumatic facial paralysis.

In conclusion, the present available trials provide limited evidence on the effectiveness of NGF in the treatment of Bell’s palsy. The number and quality of trials are too low to draw firm conclusions. Further rigorous RCTs that can overcome the numerous limitations of the current evidence are required.

## Figures and Tables

**Table I:** Summary of clinical studies of NGF for Bell’s palsy.

First author, year (reference)	Age in years	Gender (M/F)	Intervention group NGF regime^a^	Control group drug therapy regime	Main outcome/days from onset	Jadad score
Zhang, 2006 (14)	A: 28.5±10.6 B: 28.7±8.9	32/13 35/17	4 μg i.m. q.d. 28 days (n=45)	p.o.: Prednisone 1 mg/kg for 3 days tapered to 5 mg/day, 15 days; vitamin B6 + fursultiamine + mecobalamin for 4 weeks (n=52)	H-B score/28	2
Tang, 2008 (15)	38±8^b^	52/42	4 μg i.m. q.d. 21 days (n=48)	p.o.: Prednisone 1 mg/kg for 3 days tapered to 5 mg/day, 21 days; mecobalamin for 3 weeks (n=46)	H-B score/21	2
Zhang, 2010 (16)	A: 25.5±10.6 B: 28.7±8.9	19/11 21/9	4 μg i.m. q.d. 28 days (n=30)	p.o.: Prednisone 1 mg/kg for 3 days tapered to 5 mg/day, 15 days; vitamin B6 + fursultiamine + mecobalamin for 4 weeks (n=30)	H-B score/28	2
Mo, 2011 (17)	A: 45.3±3.4 B: 43.4±4.2	18/12 20/10	4 μg i.m. q.d. 7 days (n=30)	i.v. drip: Dexamethasone 10 mg for 7 days + Danhong injection 20 ml for 7 days; p.o.: Mecobalamin for 7 days; physical therapy for 20 days (n=30)	H-B score/30	2
Liu, 2012 (18)	A: 45.3±3.4 B: 43.4±4.2	18/10 20/10	30 μg i.m. q.d. 7 days (n=28)	i.v. drip: Dexamethasone 10 mg for 7 days + PNS 300 mg for 7 days; p.o.: Vitamin B1 + mecobalamin for 7 days; physical therapy for 21 days (n=30)	H-B score/30	3
Wang, 2012 (19)	A: 43.7±14.9 B: 42.5±15.2	29/28 31/27	30 μg i.m. q.d. 7 days (n=57)	p.o.: Prednisone 1 mg/kg for 3 days tapered to 5 mg/day, 30 days; i.m.: Vitamin B1 + mecobalamin q.d. for 30 days (n=58)	H-B score/28 ENoG/28	3
Li, 2013 (20)	A: 48.6±13.7 B: 48.9±14.5	25/24 26/23	30 μg i.m. q.d. 7 days (n=30)	i.v. drip: Acyclovir 500 mg b.i.d. for 7 days; p.o.: Prednisone 1 mg/kg for 3 days tapered to 5 mg/day, 15 days; i.m.: Vitamin B1+ vitamin B12 for 7 days (n=30)	H-B score/28	2
Tian, 2013 (21)	A: 29.6±12.3 B: 34.7±11.2	c c	30 μg i.m. q.d. 7 days (n=49)	i.v.drip: Dexamethasone 10 mg for 7 days; i.m.: Vitamin B1+ vitamin B12 for 7 days (n=49)	H-B score/28 ENoG/28	2

Age is presented as mean ± standard deviation.

a The intervention group also received the drug therapy regime administered to the control group.

b Separate data for groups A and B not available.

c Data not available.

A, intervention group; B, control group; q.d., once a day; b.i.d., twice a day; i.m., intramuscular injection; i.v. drip, intravenous drip; p.o., per oral; PNS, *Panax notoginseng* saponins; H-B score, House-Brackmann Facial Recovery scale; ENoG, electroneurography; NGF, nerve growth factor; M, male; F, female.
